# Sexual dysfunction in infertile couples

**DOI:** 10.1192/j.eurpsy.2021.1467

**Published:** 2021-08-13

**Authors:** M. Daoud, S. Omri, N. Smaoui, R. Feki, M. Maalej Bouali, L. Zouari, J. Ben Thabet, N. Charfi, M. Maalej

**Affiliations:** 1 Department Of Psychiatry “c”, Universty Hospital of Hedi Chaker, sfax, Tunisia; 2 Psychiatry C Department, Hedi chaker University hospital, sfax, Tunisia

**Keywords:** sexuality, infertility, woman, Dysfonction

## Abstract

**Introduction:**

Interactions between infertility and sexuality are numerous and complex. Recently more attention is being paid to the impact of infertility on the marital sexuality.

**Objectives:**

The aim of this study was to determine the effects of infertility on sexual functions.

**Methods:**

A cross-sectional descriptive study, the obstetric gynecology department Basic demographic information was collected. Respondents were surveyed regarding sexual impact and perception of their infertility etiology.

**Results:**

Our patients had an average age of 33.2. The average number of years of infertility was 3.9 years.. The most common cause of female infertility was an ovulat disorder (36%), that of male infertility was sperm production defect. The confrontation with a diagnosis of infertility marks a difference in the way couples organize their sexual life. In our study, sexual problems after this diagnosis were experienced by 38% of women. Sexual dysfunction was detected as a pain problem (24%), a desire problem (10%), an arousal problem (4%), and an orgasm problem in 6% and. Faced with this situation, women felt guilty (46%), angry (72%) and anxious (82%). Infertility was perceived as the worst experience of life by 78% of our patients.

**Conclusions:**

Infertility can interfere negatively in women sexuality. The investigation of sexual difficulties in infertility consultations must be systematic.

